# *In vitro* and *in vivo* properties differ among liquid intravenous immunoglobulin preparations

**DOI:** 10.1111/j.1423-0410.2012.01648.x

**Published:** 2013-02

**Authors:** F Dhainaut, P-O Guillaumat, H Dib, G Perret, A Sauger, C de Coupade, M Beaudet, M Elzaabi, L Mouthon

**Affiliations:** 1 LFB BiotechnologiesCourtaboeuf, France; 2 Université Paris Descartes, Faculté de Médecine, Institut CochinINSERM U1016, Paris, France; 3 Université Paris Descartes, Pôle de Médecine Interne, hôpital Cochin, Centre de Référence pour les vascularites nécrosantes et la sclérodermie systémiqueAssistance Publique-Hôpitaux de Paris, Paris

**Keywords:** intravenous immunoglobulin, repertoires, liquid, factor XI, IgA, complement activation

## Abstract

**Objective** To compare *in vitro* and *in vivo* biological and biochemical properties of five liquid intravenous immunoglobulin (IVIg) preparations licensed for therapeutic use in Europe.

**Methods** ClairYg® was compared in a blinded manner to four other liquid IVIg preparations licensed in Europe (Octagam®, Kiovig®, Gamunex®, Privigen®). Three batches of each preparation were tested, except for the IgG repertoires and the animal model.

**Results** Levels of anti-A and anti-B antibodies were lower in ClairYg® (0·11/0·11) relative to a positive EDQM standard and Octagam® (0·11/0·08) than in other preparations (0·33–0·69/0·42–0·46). IgG in ClairYg® recognized 365 and 416 protein spots in HEp-2 cell and *Escherichia coli* protein extracts vs. 230–330 and 402–842 protein spots, respectively, for IgG in other preparations. IgA content (301 vs. 165–820 ng/mg of IgG), Factor XI and Factor XII antigen (0·46 vs. 0·85–2·40 mU/mg of IgG and 7·8 vs. 20·0–46·2 lU/mg of IgG) C1q binding (0·42 vs. 0·67–1·89 arbitrary units) and C5a uptake (0·41 vs. 0·45–0·66% of activation) were lower in ClairYg® than in other preparations. Finally, intravenous infusion of ClairYg®, Gamunex® and Privigen® had no major effect on arterial blood pressure in spontaneously hypertensive rats.

**Conclusions** Our results evidence some differences in the biological and biochemical properties among licensed liquid IVIg preparations.

## Introduction

Intravenous immunoglobulin (IVIg) are therapeutic preparations of normal human IgG obtained from pools of 20 000 to 40 000 healthy blood donors and thus contain a wide range of IgG reactivities [1]. Initially used as a replacement therapy for patients with primary and secondary immune deficiencies, IVIg preparations are now also used for the treatment of a number of autoimmune and/or systemic inflammatory diseases [2, 3]. The large donor pool ensures the diversity of IgG repertoire, and IVIg preparations are composed of a broad range of immune antibodies directed against pathogens and foreign antigens, natural autoantibodies and also anti-idiotypic antibodies [4].

An ideal immunoglobulin (Ig) preparation intended for clinical use should be safe and efficacious. Efficacy depends on suitable levels of protective antibodies against pathogens and the functional integrity of the Ig molecule. Plasma products for therapeutic use pose specific challenges in manufacturing to ensure biological activity and safety with respect to potential contamination and transmission of disease-causing agents. As the manufacturing process, virus reduction methods, and final formulation and composition differ widely among these products, it is intuitive that safety and efficacy could also differ [5].

Intravenous immunoglobulin infusions are in most cases very well tolerated. Minor adverse effects such as hypertension, fever and chills, nausea, myalgias or headache are reported during IVIg infusion [6]. Severe complications are rare and include anaphylaxis, mainly due to immunization against IgA in patients with IgA deficiency, haemolysis due to the presence of haemagglutinins in IVIg preparations [7], acute renal failure [8], stroke and myocardial infarction [9]. Tolerance is actually a major issue, and a number of procedures have been proposed to improve the tolerance of IVIg preparations. However, very few studies are available that compare IVIg preparations obtained through different manufacturing processes for the presence of co-purified proteins and/or activation of biological cascades.

In the present work, we compared in a blinded manner three batches of five liquid IVIg preparations licensed in Europe including ClairYg®, a new liquid IVIg preparation obtained through ethanolic and caprylic acid precipitations [10], and provide evidence for some differences in the biological and biochemical properties among IVIg preparations tested.

## Materials and methods

### Intravenous immunoglobulin fractionation process

ClairYg® is a ready-to-use 5% liquid formulated polyvalent human IgG for intravenous administration fulfilling the current Note for Guidance on the Clinical Investigation of IVIg (CPMP/BPWG/94033/2007 rev.2) and the Core Summary of Product Characteristics for IVIg (CPMP/BPWG/94038/2007 rev.3). The purification of IgG from plasma is based on ethanolic and caprylic acid precipitations [10, 11]. To inactivate enveloped viruses, the protein solution is submitted to a solvent/detergent treatment. Then, IgG are purified by a succession of two chromatography steps. First, an anion exchange chromatography (Trimethyl-amoniumethyl chromatography) is performed, followed by an affinity chromatography that allows for the depletion of anti-A and anti-B haemagglutinins. To ensure viral safety, a second dedicated step, nanofiltration through a 20 nm pore size filter (Planova; Asahi Kasei Bioprocess, Tokyo, Japan), at acidic pH is performed. Finally, the IgG solution is stabilized in mannitol, glycine and polysorbate 80, adjusted at pH 4·8 and sterile-filtered before filling. Fractionation processes and virus removal steps used for other liquid IVIg preparations, Octagam® [12], Kiovig® [13], Gamunex® [14], Privigen® [15] are indicated in Table 1.

**Table 1:** Fractionation processes used for the five liquid intravenous immunoglobulin preparations tested

Product	Plasma fractionation	Ion exchange step	Viral inactivation	Protein concentration, g/l	Formulation
Clayrig®	Ethanol/caprylate	Yes	SD + nanofiltration	50	Liquid pH 4·8
Octagam®	Ethanol	No	SD + acidic pH	50	Liquid acidic pH
Kiovig®	Ethanol	Yes	SD + nanofiltration + acidic pH	100	Liquid acidic pH
Privigen®	Ethanol/caprylate	Yes	SD + nanofiltration + acidic pH	100	Liquid acidic pH
Gamunex®	Ethanol/caprylate	Yes	Caprylate + acidic pH	100	Liquid acidic pH

SD, solvent/detergent.

### Experimental procedure

In all experiments performed, except for the analysis of IgG antibody repertoires and assessment of haemodynamic effects of IVIg preparations in a rat model, three different batches of each IVIg preparation were tested. ClairYg® was compared in a blinded manner to four other liquid IVIg preparations licensed in Europe including Octagam®, Kiovig®, Gamunex® and Privigen®. The code was broken after realization of the last experiment. IVIg concentration was 5% for two preparations and 10% in the three others (Table 1). The same protein concentration was used in all experiments.

### Quantification of IgG in intravenous immunoglobulin preparations

Concentration of IgG in each batch was obtained by nephelometry using the standard method provided by Siemens (BNII, Saint Denis, France).

### Quantification of IgA in intravenous immunoglobulin preparations by enzyme-linked immunosorbent assay

Flat-bottom microtitre plates (Dynex Technologies, ref: M129B, Chantilly, VA, USA) were coated with anti-alpha chains human IgA rabbit polyclonal antibodies (Dako, Glostrup, Denmark) in phosphate-buffered saline (PBS) overnight at 4°C. After saturation with PBS + 1% bovine serum albumin (BSA from Sigma-Aldrich, ref A7030 Saint Louis, MO, USA), samples and normal human serum (Siemens Healthcare, Marburg, Germany) diluted in PBS + 0·1% Tween (Merck, ref: 8.22184.0500, Darmstadt, Germany) (PBS-Tween) + 0·1% BSA were incubated 2 h at 37°C. Then, a peroxidase-conjugated rabbit anti-human IgA antibody (Dako) was used as a secondary antibody and incubated 2 h at 37°C. After washing in PBS-Tween, *O*-Phenylene-Diamine (OPD) (Sigma-Aldrich, ref: P-8412) was added in citrate–phosphate buffer at pH 5·0 for 2 min and then stopped with 2 m H_2_SO_4_. Optical density was measured at 490 nm. IgA concentration was determined in each batch and the results were expressed as ng of IgA per mg of IgG.

### Dosage of monomers, dimers, polymers and fragments

Measurements of fragment, monomer, dimer and polymer concentrations were performed using the European Pharmacopeia method 01/2009: 0918.

### Dosage of haemagglutinins in intravenous immunoglobulin preparations

#### Flow cytometry

Red blood cells (RBC) were collected on ethylenediaminetetraacetic acid (EDTA) from patients belonging to A or B groups, all of them being Rh(D) negative. RBC were washed twice in NaCl 0·9% (Braun Medical ref: 0066570E Boulogne Billancourt, France) (centrifugation at 1730 ***g*** for 5 min in between washes). Two million RBC were distributed in a U-bottom microtitre plate (Greiner Bio-One, Ref: 650101, Kremsmünster, Austria) and 50 μl of a standard curve IVIg using an immunoglobulin positive control (EDQM n° Y0001152 : maximum permissible pharmacopoeial level in haemagglutination tests, Strasbourg, France) [16] or the diluted samples at a working concentration (in PBS pH 7·4 with 1% BSA) were added. Plates were incubated for 2 h at 37°C under shaking. Wash steps in PBS-BSA were repeated (3 × 1 min at 770 g). A goat F(ab’)_2_ anti-human IgG (Fc) phycoerythrin-conjugate (Beckman Coulter, Ref: PN IM0550, Villepinte, France) was used at a dilution of 1/20 in PBS-BSA. Plates were incubated 30 min at room temperature and protected from light. Wash steps were performed as described above. Each pellet was resuspended in 200 μl of PBS-BSA, and read with a Beckman Coulter Cytomics FC 500 flow cytometer (Beckman Coulter). The mean fluorescence intensity (MFI) of the positive control IVIg was plotted against IgG concentration (standard curve) for concentrations ranging from 0·23 to 30 g/l. Results were expressed as the ratio between the sample line slope and the positive control IVIg standard line slope. The standard curve equation is *y* = *ax* + *b*, where ‘*a*’ is the slope value of the standard line and ‘*b*’ is the zero point corresponding to the assay background. As the sample equation is *y*′ = *a*′*x* + *b* and using known values of the sample’s MFI (*y*′) and IgG concentration (*x*′), the slope ratio was calculated as [(MFI-b)/(IgG concentration)]/*a*.

#### Haemolytic activity based on flow cytometry cell counts

Peripheral blood was collected from patients of group A, B, AB and O, all Rh(D) negative, on heparin and washed twice in NaCl 0·9% (centrifugation at 1730 ***g*** for 5 min in between washes). RBC pellets were treated with papain according to the manufacturer instructions (Bio-Rad, Ref: 86594, Marnes-La-Coquette, France).

To ensure a good correlation between cell counts and viability, cells were stained with calcein acetoxymethyl ester (calcein-AM; Invitrogen-Fischer Bioblock Ref: C3099, Illkirch, France) for 30 min at 37°C under shaking. After washing twice for 5 min at 480 g, U-bottom microtitre plates (Greiner Bio-One, Ref: 650101) were saturated with PBS, 1% BSA (Sigma-Aldrich, ref: A7030) (30 min at room temperature) and 2·5 × 10^5^ calcein-AM-red RBC were incubated in the presence of 50 μl of four IVIg dilutions (concentration ranging from 2 to 40 g/l). A standard curve using a positive IVIg international reference (EDQM n° Y0001152) [16] was added as well as internal controls, including 50 μl of cell suspension incubated either with 150 μl of water for injectable preparation for the total cell lysis or with 50 μl of PBS pH 7·4, 1% (w/v) BSA for spontaneous lysis (RBC lysis during the assay without addition of any IVIg) or 50 μl of O+ serum tested as a positive internal control of the haemolytic reaction. Cells were incubated for 45 min at +37°C under shaking. Then, 100 μl of guinea-pig complement (Tebu-Bio, Ref: C300-0010, Le Perray en Yvelines, France) was added at working concentration in each well, except for the total cell lysis condition, and 1-h incubation at +37°C with shaking was performed before centrifugation (5 min at 480 ***g*** room temperature). RBC were analysed on a Beckman Coulter Cytomics FC 500 flow cytometer (Beckman Coulter). Results were expressed as a percentage of specific cell lysis.

### IgG antibody repertoires study

#### HEp-2 cell culture and protein extraction

HEp-2 cells, a cell line derived from a human laryngeal carcinoma that represents the standard substrate for the detection of antinuclear antibodies [17], were obtained from EuroBio (Les Ulis, France) and cultured as described [18, 19]. Confluent cells were detached by use of 0·05% trypsin-EDTA (Gibco BRL, Invitrogen, Grand Island, NY, USA) and then washed twice with PBS and once in TBS (25 mm Tris pH 7·5, 138 mm NaCl, 2·7 mm KCl). Protein extraction with enrichment in nuclear proteins was performed as previously described [20, 21].

#### *Escherichia coli* culture and protein extraction

*Escherichia coli* bacteria CIP 54·127 were cultured in Tryptic Soy Broth (Soybean-Casein Digest) medium (BD Bacto, Franklin Lakes, NJ, USA). A pellet of 19 billion bacteria was homogenized in a buffer containing 5 m urea, 2 m thiourea, 2% (w/v) 3-[(3-cholamidopropyl)dimethylamino]-1-propane sulfonate, 2% (w/v) SB 3–10, 40 mm Tris, 0·2% Bio-Lyte 3/10 and gently poured over V/V of 0·1-mm glass beads (BioSpec Products, Bartlesville, OK, USA) and then vigorously vortexed for 4 min. The supernatant was then ultra-centrifuged twice at 150 000 **g**, 25 min at 4°C. Proteins were quantified using the Bradford method and then extracts were aliquoted and frozen at −80°C until use.

#### Two-dimension electrophoresis, electrotransfer and immunoblotting

We used pH 3–10 and acrylamide gradient of 7–18% in all experiments, which allowed for studying a wide range of antigens of 10–250 kDa [22, 23]. Two-dimension gel electrophoresis, electrotransfer, immunoblotting, gel staining and image analysis of gels and 2-D blots were performed as recently reported [22, 24].

### Complement activation in intravenous immunoglobulin preparations

#### Evaluation of intravenous immunoglobulin binding to C1q

Intravenous immunoglobulin binding to C1q was performed according to [25]. Briefly, flat-bottom low IgG affinity microtitre plates were incubated with 2 μg/ml human C1q (Calbiochem Ref: 204876 Fontenay sous bois, France), diluted in 0·1 m bicarbonate pH 9·6 buffer for 1 h at room temperature and then overnight at +4°C. After washing (3 × 5 min using PBS-Tween 0·1%), 300 μl of PBS pH 7·4 and 1% BSA (Sigma-Aldrich, ref: A7030) were incubated 1 h at room temperature. The washing procedure was repeated as described above and 100 μl of each sample dilution was incubated for 60 min at room temperature followed by 90-min incubation at +37°C. A total of eight different concentrations per sample were analysed starting from 1 g/l (1:2 dilutions in sample buffer: PBS-Tween 0·1%, 0·1% BSA, 0·1 m NaCl). After washing (3 × 5 min using PBS-Tween 0·1%), 100 μl of horse radish peroxidase (HRP) goat anti-human IgG (Fc γ-chain specific) conjugate (Jackson ImmunoResearch Ref: 109-035-098, West Grove, PA, USA) was incubated for 90 min at +37°C. After washing, revelation was stopped after 4–7-min incubation at room temperature with OPD (Sigma Ref: P8412) substrate, using 50 μl of 4 m NH_2_SO_4_. Results were expressed as a ratio between OD 492 units of the samples and the OD 492 nm of a human Ig biological reference preparation A (BRP A) reference, a Ph. Eur. reference standard (EDQM, catalogue code: H0990000, BRP batch 3).

#### Complement activation assay by C5a release

Human serum was obtained by pooling the serum of four healthy donors (AB group) and stored at −80°C. Before use, the serum was gently thawed at 4°C. Twenty μl of tested Ig at a concentration of 50 mg/ml was added to 80 μl of ¼ diluted serum in PBS + 1% BSA (Jackson ImmunoResearch, Ref: 001-000-162). All test tubes were kept in a water-ice mix up to the incubation. For each sample, five independent C5a activation tests were performed. Maximum complement activation was obtained by replacing the tested Ig by 10 mg/ml of *E. coli* LPS (Sigma-Aldrich, ref: L3880). A positive control was performed with thermally aggregated Ig in the same concentration. A negative control was performed with the PBS-BSA buffer. The mixture was incubated 1 h at 37°C and the reaction was stopped by putting the test tubes in ice and by adding 1 μl of 100 mm EDTA (Sigma-Aldrich Ref: E5134). After the incubation, C5a release in each test tube was assayed in triplicate by enzyme-linked immunosorbent assay (ELISA) method as previously described [26]. Flat-bottom microtitre plates were coated with 100 μl of anti-C5a (R&D systems Ref: MAB 2037, Lille, France) at 0·4 μg/ml in carbonate buffer 0·1 m pH 9·6 overnight at 4°C. After saturation with PBS + 1% BSA, samples and controls were diluted in saturation buffer, deposited in the plate and incubated 2 h at 20°C. The polyclonal biotinylated antibody (R&D systems Ref: BAF 2037) was then added and revealed with a peroxidase-conjugated streptavidin (DY998; R&D Systems). The absorbance was measured at 492 nm using an ELISA reader (Infinite M200; Tecan, Lyon, France). The percentage of complement activation was obtained according to the following formula: (Sample-Negative control)/(Maximum complement activation − Negative control) × 100. For each sample, the result corresponds to the mean obtained on the five independent activations. Acceptance criteria were applied: the positive control should be between 90% and 110% with an OD close to 1·3.

### Dosage of Factor XI and Factor XII antigens by enzyme-linked immunosorbent assay

Flat-bottom microtitre plates were coated with goat anti-FXI or anti-FXII polyclonal antibodies (Cedarlane, Burlington, Ontario, Canada) in carbonate buffer 0·1 m pH 9·6 overnight at 4°C. After saturation with PBS + 2% BSA (Sigma-Aldrich ref: A7030), IVIg preparations and standard (human plasma Unicalibrator, Stago, Asnières, France) diluted in PBS-Tween 0·1% (Merck ref: 8·22184·0500) + 0·1% BSA for FXI or + 2% BSA for FXII were incubated 1 h 30 min at 20°C. Then, peroxidase-conjugated anti-FXI or anti-FXII goat polyclonal antibodies were incubated 1 h 30 at 20°C. After washing in PBS-Tween, OPD (Sigma-Aldrich ref: P-8412) was added in citrate–phosphate buffer at pH 5·0 for 2 min 30 s and then stopped with 2 m H_2_SO_4_. Optical density was measured at 490 nm. FXI Ag or FXII Ag concentrations were determined in each batch, and the results were expressed as ng of FXI or FXII per mg of IgG.

### Assessment of haemodynamic effects of different intravenous immunoglobulin preparations in a rat model

The experiments were carried out using 42 14–15-week-old male SHR (Centre d’Elevage R. Janvier, B.P. 55, Le Genest-Saint-Isle, France). Hypertensive rats were anesthetized with sodium pentobarbital (60 mg/kg i.p.). After endotracheal intubation, rats were artificially ventilated (approximately 1 ml/100 g body weight and 60 strokes/min) with ambient air enriched with oxygen such as to maintain blood gas parameters (PO_2_, PCO_2_, pH) within physiological limits. One carotid artery and the penile vein were catheterized for blood pressure measurement and drug administration, respectively. Body temperature was maintained at 37–38°C by means of a heating pad.

The heparin-filled (100 IU/ml) catheter in the carotid artery was connected to a strain gauge manometer (model DT-XX, Ohmeda; GE Healthcare, Limonest, France) precalibrated with a mercury manometer.

Following an equilibration period of at least 15 min, necessary to obtain stable hemodynamic conditions, intravenous administration of each test/reference item was performed. Rats were injected either with the formulation buffer or one of the five IVIg preparations in a blinded manner. Nicardipine was obtained from Sigma. Haemodynamic parameters were continuously monitored and digitized at a sampling rate of 250–1000 Hz in each channel using hem software (Notocord Systems, Croissy-Sur-Seine, France) and stored on the hard disk of a personal computer. Most of the experiments followed the same general procedures as described in [27].

Treatments were administered as an intravenous infusion under the following conditions (amendment to study Plan no.1): IVIg preparations concentrated at 10% and the formulation buffer were administered at 3·33 ml/kg/min over 3 min, whereas IVIg preparations concentrated at 5% were administered at 3·33 ml/kg/min over 6 min. The treatments above were blinded and included one control item group (formulation buffer) and five test item groups treated at 1000 mg/kg. Treatment unblinding was made at the end of the experiments. As a positive reference item group, six animals are treated with an intravenous infusion (2 ml/kg/min over 3 min) of nicardipine 30 μg/kg.

The intravenous route of administration was used to evaluate the cardiovascular effects of the different compounds since it is the intended clinical route.

The following parameters were measured: systolic arterial pressure (SAP, mmHg); diastolic arterial pressure (mmHg); mean arterial pressure (MAP, mmHg); heart rate (HR, beats/min). Haemodynamic parameters were measured at baseline (before injection, *T*_0_), at 3 (*T*_3_), 6 (*T*_6_), 10 (*T*_10_), 15 (*T*_15_), 30 (*T*_30_) and 45 (*T*_45_) min after the beginning of the intravenous infusion.

### Statistical analysis

Data were presented using descriptive statistics (*n*, mean, standard deviation (SD), SEM, minimum, maximum) for raw data, and changes from baseline (delta and delta %) and graphs illustrating means (±1 SEM) of each group over time were provided. In the case of experimental data obtained in rats, for each parameter, homogeneity of baseline values (*T*_0_) between the seven groups was tested using a one-way anova. For each parameter, changes from baseline value (delta) at each measurement time were compared using a two-way anova (group, time) with repeated measurements over time.

## Results

### Anti-A and anti-B haemagglutinins

**Figure 1:**
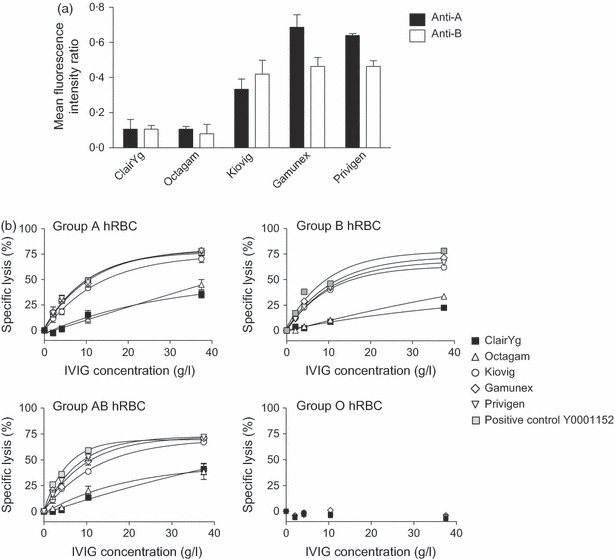
(a) Quantification of anti-A (black box) and anti-B (white box) haemagglutinins in five liquid intravenous immunoglobulin (IVIg) products tested in triplicates at 0·23–30 g/l using flow cytometry assay. Data represent the fluorescence intensity ratio [mean ± standard deviation (SD)] between each sample line slope and positive control from EDQM (Y0001152). (b) Specific lysis of red blood cells from A, B, AB and O Rh(D) negative groups induced by five liquid IVIg products (ClairYg® (▪), Octagam® (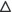), Kiovig® (○), Gamunex® (◊), Privigen® (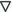) or [positive *c*ontrol (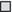)] in the presence of guinea-pig complement.

ClairYg® and Octagam® had a low level of anti-A and anti-B haemagglutinins, whereas other IVIg preparations tested expressed threefold to sixfold higher levels of anti-A and anti-B haemagglutinins (Fig. 1a). Specific lysis of human RBC from A, B and AB Rh(D) negative groups varied according to the amount of IVIg added, whereas no lysis occurred with human RBC from O group (Fig. 1b). Again, with all three types of RBC, a lower level of haemolysis was observed with ClairYg® and Octagam®, than with the other three IVIg preparations (Fig. 1b).

### Analysis of IgG antibody repertoires in intravenous immunoglobulin preparations

**Figure 2:**
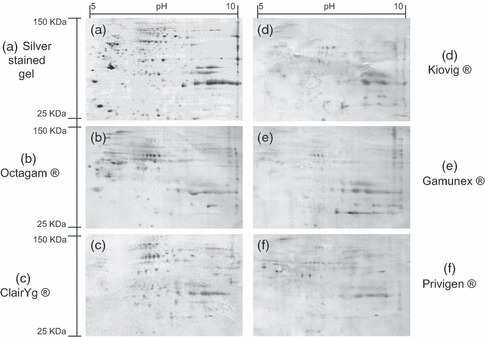
Two-dimension immunoblotting of IgG preparations reacting with HEp-2 cell proteins. Silver-stained gel of HEp-2 cell proteins (a); HEp-2 cell proteins recognized by immunoblotted IgG from five different intravenous immunoglobulin preparations: Octagam® (b), ClairYg® (c), Kiovig® (d), Gamunex® (e) and Privigen® (f) tested at 100 μg/ml. *x*-axis: pH range 5–8; *y*-axis: range of molecular weight 150–25 kDa.

**Figure 3:**
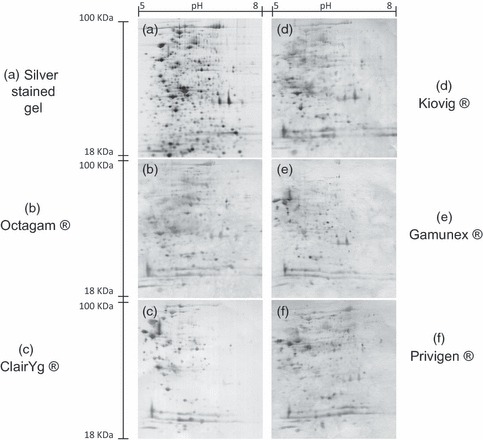
Two-dimension immunoblotting of IgG preparations reacting with an *Escherichia coli* protein extract. Silver-stained gel of *E. coli* protein extract (a); *E. coli* proteins recognized by immunoblotted IgG from five different intravenous immunoglobulin preparations: Octagam® (b), ClairYg® (c), Kiovig® (d), Gamunex® (e) and Privigen® (f) tested at 100 μg/ml. *x*-axis: pH range 5–8; *y*-axis: range of molecular weight 100–18 kDa.

The five IVIg preparations were tested with a protein extract of HEp-2 cells enriched with nuclear proteins (Fig. 2). IgG in ClairYg® recognized 365 protein spots vs. 230–330 protein spots, respectively, for IgG in other preparations (Table 2). In fact, Octagam® and at a lower level Privigen® recognized a greater number of protein spots in isoelectric pH between five and seven, and Octagam® and ClairYg® recognized a higher number of proteins of high molecular weight than other preparations. The five IVIg preparations were also tested for IgG reactivity with a protein extract of *E. coli* (Fig. 3). IgG in ClairYg® recognized 416 protein spots vs. 402–842 protein spots for IgG in other preparations (Table 2). ClairYg® recognized a lower number of protein spots of high isoelectric pH than other IVIg preparations, whereas Octagam® expressed a lower number of high reactivity spots in the low isoelectric pH than other IVIg preparations (Fig. 3). Thus, the repertoires of IgG reactivities were markedly heterogeneous among the five IVIg preparations tested.

**Table 2:** Number of IgG reactivities expressed in different intravenous immunoglobulin (IVIg) preparation towards Hep-2 cells and *Escherichia coli* antigens

IVIg preparations	Source of antigens	
HEp-2	*E. coli*
ClairYg®	365	416
Octagam®	330	653
Kiovig®	288	842
Gamunex®	321	402
Privigen®	230	458

### Quantification of IgA in intravenous immunoglobulin preparations

Quantification of IgA varied widely among the five IVIg preparations tested. Privigen® had a lower level of IgA compared with ClairYg® and Gamunex®, whereas IgA content was higher in Kiovig® and Octagam® (Table 3).

**Figure 3:** Comparison of biological and biochemical properties among five licensed liquid intravenous immunoglobulin preparations

Components	Anti-A^a^	Anti-B^a^	IgA (ng/mg IgG)	Polymers (%)	Dimers (%)	Monomers (%)	Fragments (%)	C1q (AU)	C5a (%)^b^
ClairYg®	0·11 ± 0·03	0·11 ± 0·01	301 ± 148	1·3 ± 0·2	7·0 ± 0·3	91·2 ± 0·1	0·5 ± 0·1	0·42 ± 0·04	14 ± 1·5
Octagam®	0·11 ± 0·01	0·08 ± 0·03	820 ± 170	1·0 ± 0·1	6·8 ± 1·4	91·6 ± 1·4	0·6 ± 0·1	1·66 ± 0·14	32 ± 1·7
Kiovig®	0·33 ± 0·03	0·42 ± 0·05	500 ± 46	1·2 ± 0·2	11·2 ± 1·3	86·8 ± 1·5	0·7 ± 0·1	0·67 ± 0·22	26 ± 1·0
Privigen®	0·64 ± 0·01	0·46 ± 0·02	165 ± 46	0·7 ± 0·1	8·8 ± 0·7	89·5 ± 0·8	1·1 ± 0·2	0·55 ± 0·11	21 ± 1·0
Gamunex®	0·69 ± 0·04	0·46 ± 0·03	385 ± 17	0·9 ± 0·1	5·1 ± 0·2	93·0 ± 0·2	1·0 ± 0·1	1·89 ± 0·37	35 ± 2·1

AU, arbitrary unit.

Data are mean ± SD (*n* = 3).

a Ratio between tested sample and EDQM positive control Y0001152.

b Percentage of activation vs. maximum complement activation using LPS.

### Dosage of monomers, dimers, polymers and fragments

Limited differences were observed among IVIg preparations regarding the content of polymers (Table 3). Kiovig® and Privigen® had the highest level of dimers, whereas Gamunex® content in dimers was the lowest. Kiovig® contained the least monomers, whereas ClairYg®, Octagam® and Gamunex® had the highest monomer content. Finally, the lowest content in fragments was detected in ClairYg® (Table 3).

### Quantification of Factor XI and Factor XII antigens by enzyme-linked immunosorbent assay

**Figure 4:**
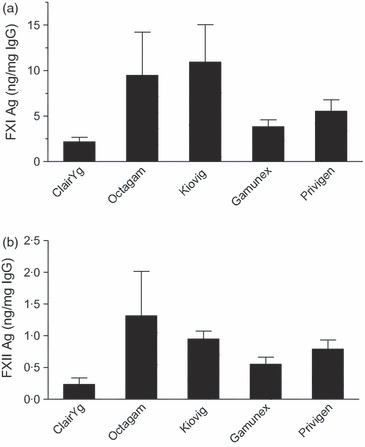
Quantification of FXI Antigen and FXII Antigen in five liquid intravenous immunoglobulin products using enzyme-linked immunosorbent assay assay. Three batches per product were tested in duplicates. Results were expressed as mean ± SD of FXI or FXII concentration in ng per mg of IgG.

Concentrations of Factor XI and Factor XII antigen varied widely among the five IVIg preparations tested. Factor XI and Factor XII antigen (0·46 vs. 0·85–2·40 mU/mg of IgG and 7·8 vs. 20·0–46·2 lU/mg of IgG with one Unit corresponding respectively to 5 and 30 μg/mL) were lower in ClairYg® than in the four other IVIg preparations (Fig. 4). The highest amounts of Factor XI and Factor XII were detected in Octagam® and Kiovig® preparations.

### Complement activation in intravenous immunoglobulin preparations

**Figure 5:**
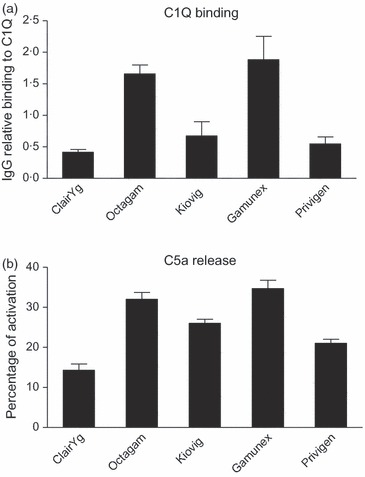
(a) Evaluation of classic complement activation pathway by C1q-binding intravenous immunoglobulin (IVIg) products. Wells were coated with human C1q and incubated with five IVIg preparations (three batches each) at eight concentration points. C1q binding was assessed using an anti-human IgG antibody (γ-chain specific). Results are expressed as the mean and SD of IVIg relative binding to C1q. (b) Evaluation of whole complement activation. After incubation of IVIg in diluted human serum at 37°C, the C5a release was assayed by enzyme-linked immunosorbent assay. Results represent the percentage of C5a release related to a maximal release obtained with LPS and are expressed as mean and SD on three batches of each IVIg.

Complement activation varied among the various IVIg preparations tested. Thus, C1q binding varied from one to almost five times, whereas C5a release varied from one to two times. C1q binding (0·42 vs. 0·55–1·89) (Fig. 5a) and C5a release (14% vs. 21–35%) (Fig. 5b) were lower in ClairYg® than in other preparations. C1q binding and C5a release were the highest in Octagam® and Gamunex®.

### Assessment of haemodynamic effects of intravenous immunoglobulin preparations in a rat model

**Figure 6:**
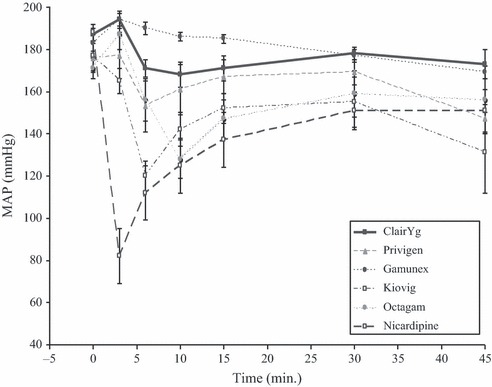
Effects of intravenous immunoglobulin (IVIg) infusion on mean blood pressure in anesthetized spontaneously hypertensive rats. Rats were injected either with the formulation buffer or one of the five IVIg preparations [ClairYg® (▪), Octagam® (•), Kiovig® (□), Gamunex® (•), Privigen® (▴)] (*n* = 5 in each case) or nicardipine (□) (*n* = 6). Mean blood pressure ± SD (mmHg) (*y*-axis) was measured at baseline (before injection), at 3, 6, 10, 15, 30 and 45 min after the beginning of the intravenous infusion (*x*-axis).

Intravenous infusion of ClairYg®, Gamunex® and Privigen® had no major effect on arterial blood pressure in spontaneously hypertensive rats whereas Octagam® and Kiovig® transiently decreased arterial blood pressure (Fig. 6). Nicardipine (30 μg/kg, intravenous infusion), used in this study as a positive reference item, displayed a clear hypotensive activity. No major effect was observed on HR by the IVIg preparation tested (data not shown).

## Discussion

In this work, we compared the biological characteristics of five liquid IVIg preparations licensed in Europe and observed biochemical differences among these IVIg preparations for anti-A and anti-B haemagglutinins, IgG antibody repertoires, IgA and factor XI and factor XII content and complement activation.

The amount of anti-A and anti-B haemagglutinins and the induction of haemolysis differed notably among the five IVIg preparations tested. Since the direct haemagglutination method still appears variable from one IVIg preparation to another and from one laboratory to another [16], we decided to use the cytometry technique applied to anti-A/B determination levels, which appears more reliable and accurate. Interestingly, ClairYg® and Octagam® had a lower level of anti-A and anti-B haemagglutinins than other IVIg preparations. The manufacturing process of ClairYg® is based essentially on caprylic acid precipitation. Since after caprylic acid precipitation anti-A and anti-B haemagglutinin levels were higher than expected, a specific affinity chromatography for anti-A and anti-B haemagglutinin depletion was added in the process, explaining the very low levels of haemagglutinins detected in ClairYg®. Interestingly, Gamunex® and Privigen®, the two other IVIg preparations using a specific caprylic acid precipitation step tested in the present work, for which there is no mention of haemagglutinin depletion in the manufacturing process, had high levels of anti-A and anti-B haemagglutinins. Although IVIg products are approved as safe and effective and meet European standards recommending low levels of anti-A and anti-B haemagglutinins (<1/64), the passive transfer of IgG antibodies against the blood group A, B and RhD antigens has been associated with adverse reactions in recipients, including haemolysis and anaemia in severe cases [7]. Since it is difficult to predict the occurrence of haemolysis, detection of haemolytic reaction could lead to reconsider continuing IVIg treatment [7, 28].

Analysis of repertoires of IgG reactivities directed at self and bacterial antigens [22, 23] identified notable differences among IVIg preparations in terms of number of IgG reactivities and target antigens recognized. Based on the results obtained with a commensal and pathogenic bacteria, *E. coli* and the cell line usually used to detect anti-nuclear antibodies, Hep-2 cells, we provide evidence that ClairYg® expresses a large range of antibody reactivities directed against bacteria or autoantigens. A number of factors can influence the antibody content of IVIg preparations including the donors that participate to the pool of plasma and the manufacturing process. Thus, depending on their genetic background, the geographic area where the donors live and the type of environmental antigens they encountered, the antibody specificities contained in IVIg preparations may vary, contributing to explain the differences in repertoires of reactivities directed at microbial antigens and/or autoantigens contained in IVIg preparations [4]. The manufacturing process may also influence IgG antibody reactivity, mainly through differences in the pH level, temperature and sodium concentration [29]. Thus, comparing IVIg preparations obtained through Cohn or propiolactone fractionation processes, we observed that the protein spots recognized by IgG in propiolactone-IVIg represented the core set of self-antigens targeted by IVIg and that an acidic pH step artificially enlarges the repertoire of self-reactive IgG [24]. However, no clinical interpretation can be made from these data, since all of the IVIg preparations tested had demonstrated efficacy in substitution and immunomodulation in patients on the basis of clinical studies despite notable differences in IgG repertoires among these IVIg preparations.

The IgA content, which potentially affects the risk and intensity of adverse events associated with administration of IVIg preparations, including anaphylactic shock, varies widely from one to another [30], depending on the purification process. For ClairYg®, the anion exchange chromatography step is dedicated to the reduction of IgA and IgM levels, although other processes such as the ability of lectin jacalin to selectively bind IgA1 subclass have been utilized in the past to deplete IgA [31].

Limited differences were observed among IVIg preparations regarding the content of dimers and polymers. Enrichment of IVIg in dimers was found to be associated with an increase in the antibody activity against self-antigens and suggests that therapeutic preparations of IVIg enriched in dimmers might exert high potential immunomodulatory activity *in vivo* [32]. However, these data remain controversial [33], mainly because it is known that the dimer level is dependent on pH formulation [34]. In addition, a number of other factors can influence the presence of fragments in IVIg preparations, among which the use of proteases such as pepsin and/or the presence of residual plasmin in the manufacturing process [35].

The manufacturing process does not only influence the antibody specificities and isotypes contained in IVIg preparations, but also the amount of non-immunoglobulin factors contained in IVIg.

Interestingly, we identified that concentrations of factor XI and factor XII antigens varied a lot between the five preparations tested, and the content in factor XI was the highest in Octagam® and Kiovig®. Interestingly, in the present work, Factor XI and Factor XII antigen concentrations were lower in ClairYg® than in the four other IVIg preparations. Because the addition of small amounts of activated factor XI to plasma can lead to production of significant amounts of thrombin, it has been suggested that activated factor XI (FXIa), which is present in IVIg preparations, could contribute to the *in vivo* risk of thrombosis after IVIg therapy [36]. In a recent work, the amount of factor XIa detected in the product correlated with the manufacturer, suggesting that variations in the manufacturing process or plasma source affect the level of factor XI in the IVIg product [36]. Importantly, the high content in factor XIa of IVIg preparations has been associated with increased risk of thromboembolic events, including stroke, pulmonary embolism, deep venous thrombosis or myocardial infarction [36, 37]. In another recent work, Kallikrein and FXIa were identified as the major contaminants with respect to coagulant activity in IVIg preparations [37]. FXIa was highly procoagulant, with the highest level in thromboembolic event–associated IVIg. Since the non-activated partial thromboplastin time unambiguously identified FXIa procoagulant activity in IVIg, its implementation as a release test would improve the safety of IVIg [37]. Although the low level of contaminating coagulation factors detected in ClairYg® may contribute to good clinical tolerance, it will need to be confirmed by the analysis of the incidence of serious adverse effect reported after IVIg injection, particularly thromboembolic events.

We have observed that complement activation varied among the various IVIg preparations tested and that C1q binding and C5a releases were lower in ClairYg® than in other preparations. The initially perceived adverse effects, stemming from complement activating aggregated IgG, including fever, chills and hypotension [38], had the effect of slowing down widespread introduction of IVIg therapy in the late 1970s, although these adverse effects have now been eliminated with amendment of the appropriate manufacturing steps. However, the capacity of different IVIg preparations to scavenge activated C3 and thereby inhibit complement activation varies between the products [39].

Finally, intravenous infusion of ClairYg®, Privigen® and Gamunex® had no major effect on blood pressure in spontaneously hypertensive rats, whereas infusion of Kiovig® and Octagam® transiently decreased arterial blood pressure. It has been proposed that polymers, complement activation and bradykinin generation might contribute to hypotension after IVIg infusion [38, 40], which is in agreement with our results in the case of Octagam® but not in the case of Gamunex®. These data provide evidence for potential heterogeneity in the induction of adverse reactions among licensed IVIg preparations.

## Conclusion

We provide evidence for the heterogeneity of biological properties including IgA, haemagglutinins, Factor XI, Factor XII concentrations, repertoires of antibodies and complement activation among five liquid IVIg preparations. These data demonstrate that the origin of plasma and the type IVIg purification processes both contribute to obtain a pharmaceutical product with distinct biochemical and physiological properties, although all IVIg preparations have demonstrated efficacy in patients with immune deficiency and systemic and/or autoimmune diseases.
